# Supplementary material for: Foldable interpenetrated metal-organic frameworks/carbon nanotubes thin film for lithium–sulfur batteries
Source: Nat Commun. 2017 Mar 6;8:14628. doi: 10.1038/ncomms14628 (PMC5343496; doi:10.1038/ncomms14628)
Supplement: Supplementary Information — Supplementary Figures 1-13, Supplementary Tables 1-5 and Supplementary References [file ncomms14628-s1.pdf]

Supplementary Information

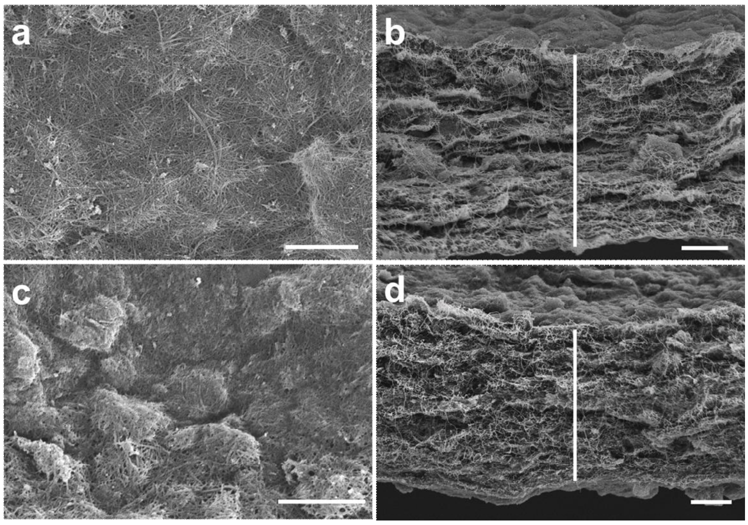

Supplementary Figure 1. Morphology and thickness characterization of MHNs/CNT thin films. (a) Surface and (b) cross-section SEM images of the CHNs/CNTs composite thin film with thickness of 8.25 μm prepared from 30 ml CHNs and 1.5 ml 0.9 mg ml^{-1}. (c) Surface and (d) cross-section SEM images of the ZHNs/CNT composite thin film prepared from 30 ml ZHNs and 1.8 ml 0.9 mg ml^{-1}. Scale bars, 1 μm (a, c), 2 μm (b, d)

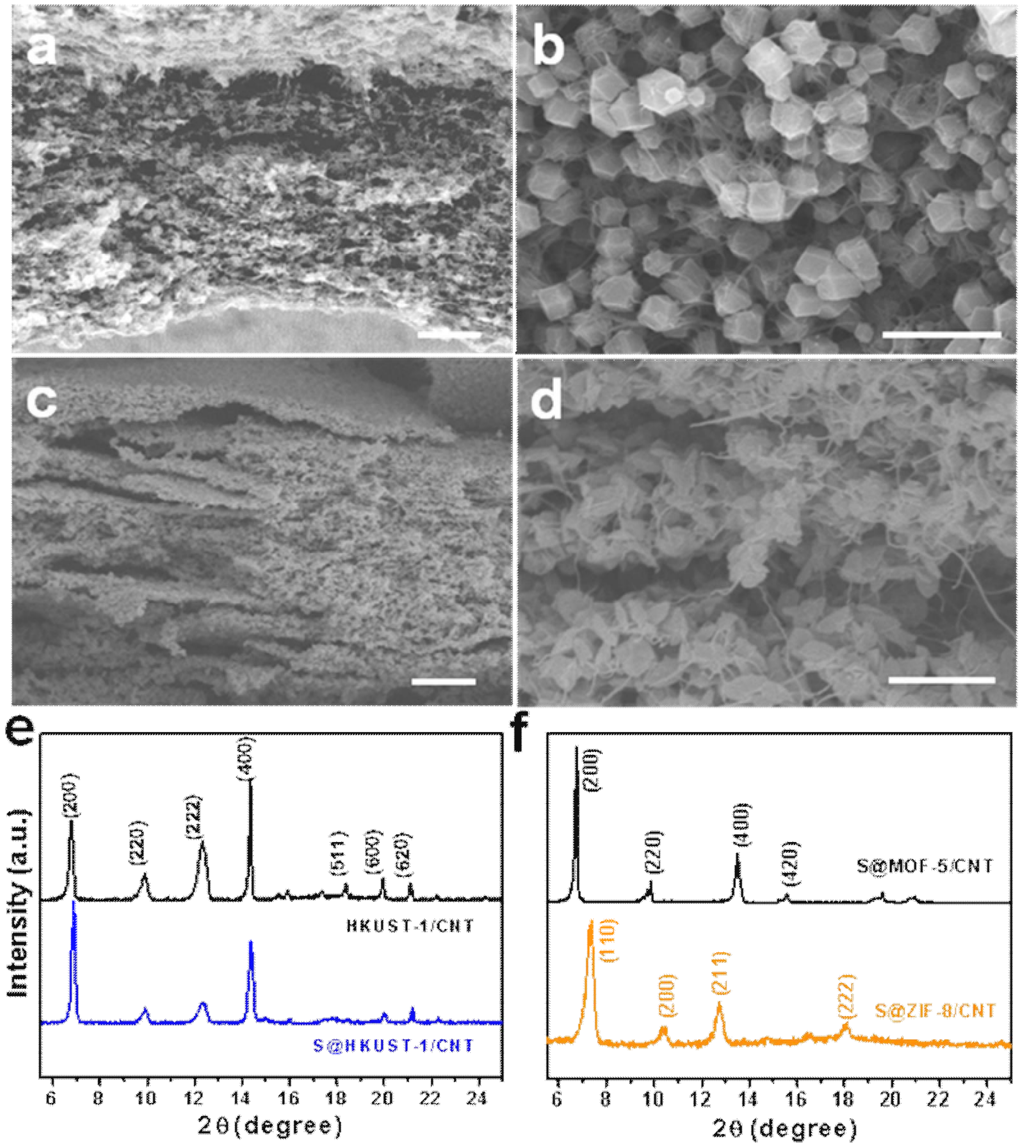

Supplementary Figure 2. Morphology and phases characterization of the prepared composite thin films. (a) Cross-section and (b) the enlarged SEM images of the flexible ZIF-8/CNTs composite thin films using 30 ml ZHNs solution mixed with 2.0 ml 0.9 mg ml^{-1} CNTs dispersion. (c) Cross-section and (d) the enlarged SEM images` of the flexible MOF-5/CNTs composite thin films using 30 ml ZHNs solution mixed with 1.8 ml 0.9 mg ml^{-1} CNTs dispersion. (e) and (f) are the XRD patterns of the prepared HKUST-1/CNT, S@HKUST-1/CNT composites and S@MOF-5/CNT, S@ZIF-8/CNT composite thin films with weight ratio of MOF to CNT of 3:2, respectively. The sulfur loading is 1 mg cm^{-2}. Scale bars, 3 μm (a, c), 1 μm (b, d).

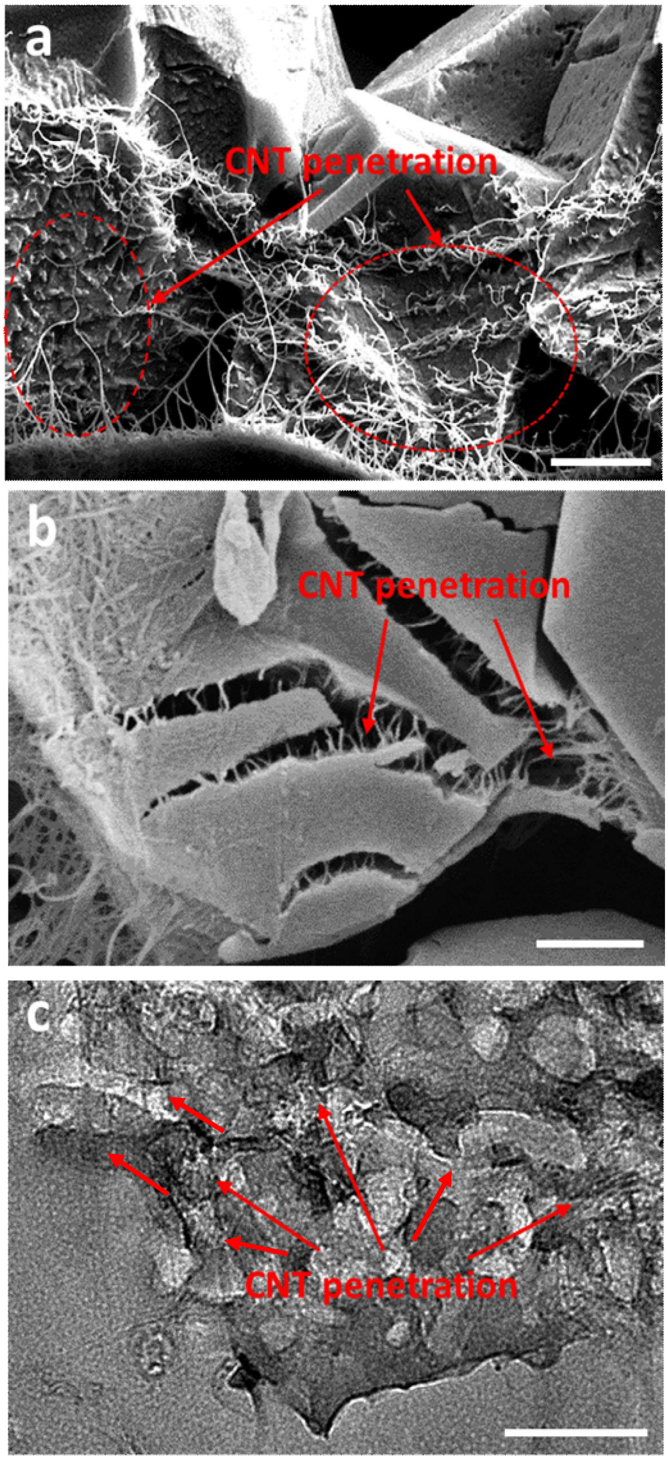

Supplementary Figure 3. The observation of CNT penetration. SEM images of the cracked HKUST-1 crystal in the as-prepared hybrid thin film (a, b) and TEM image of the carbonized hybrid thin film from HKUST-1/CNT (3:2) treated at 800 ^{o}C for 2 hours in N_2 and then washing away copper related species by diluted natric acid (10 mM). Scale bars, 1 μm (a), 500 nm (b), 100 nm (c).

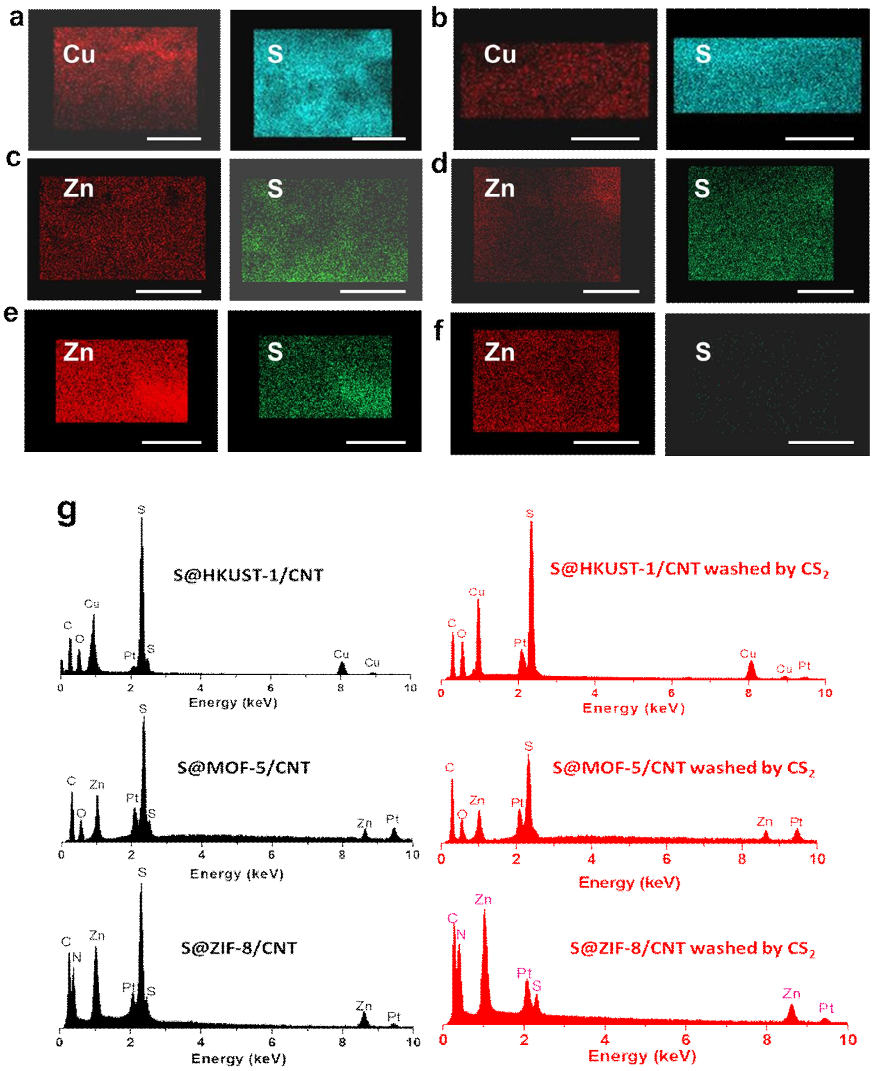

Supplementary Figure 4. Elements mapping images of S@MOFs/CNT composite films. The Cu and S distribution (a) before and (b) after washing by CS_2 for S@HKUST-1/CNT electrode; the Zn and S distribution (c) before (d) after washing by CS_2 for S@MOF-5/CNT electrode; the Zn and S distribution (e) before and (f) after washing by CS_2 for S@ZIF-8/CNT electrode, respectively. All the scale bars represent 2 μm. The ratio of MOFs to carbon is 3:2. (g) The EDX spectra of S@MOFs composite film before and after washing by CS_2. The corresponding elements weight percents in (g) are listed in Supplymentary Table 5.

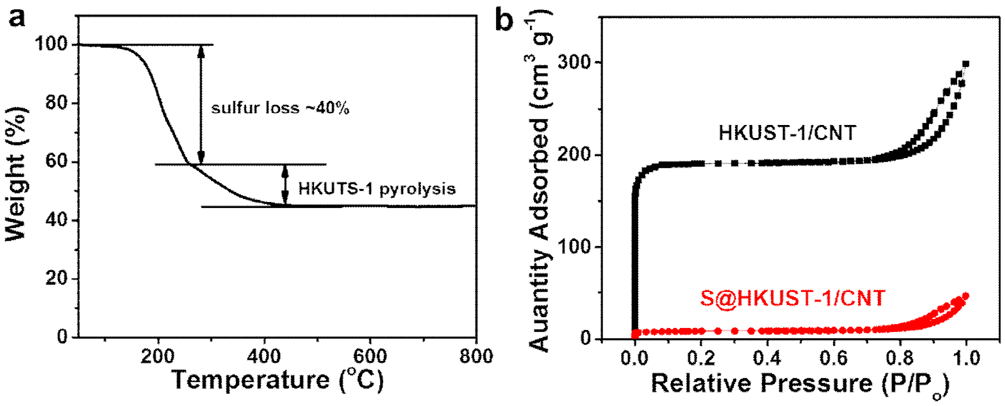

Supplementary Figure 5. (a) The TGA curve of the S@HKUST-1/CNT electrode for coin cells. (b) The N_2 adsorption/desorption isotherm of the HKUST-1/CNT thin film before and after sulfur loading. The sulfur loading is 1 mg cm^{-2}, and the weight ratio of MOFs to CNT is 3:2.

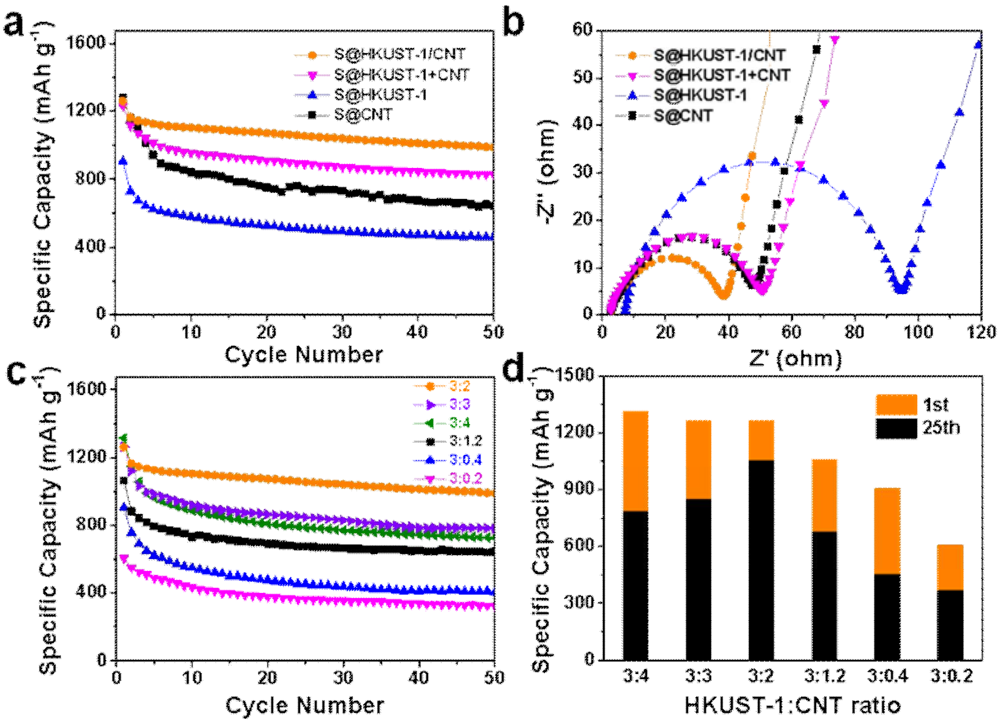

Supplementary Figure 6. Electrochemical performances of S@HKUST-1/CNT electrodes with different weight ratio of HKUST-1 to CNT. The comparison of (a) cycling performance and (b) Nyquist plots between sulfur electrodes with different matrix. The red refers to the S@HKUST-1/CNT self-standing electrode, while the black, blue and pink are S@CNT, S@HKUST-1 and S@HKUST-1+CNT (HKUST-1 and CNT are simply mixed together in weight ratio of 3:2) electrodes prepared by conventional casting process respectively. (c) The cycling performance comparison and (d) capacities variation of S@HKUST-1/CNT electrodes with the variation weight ratio of HKUST-1 to CNT. The sulfur loading is 1 mg cm^{-2}.

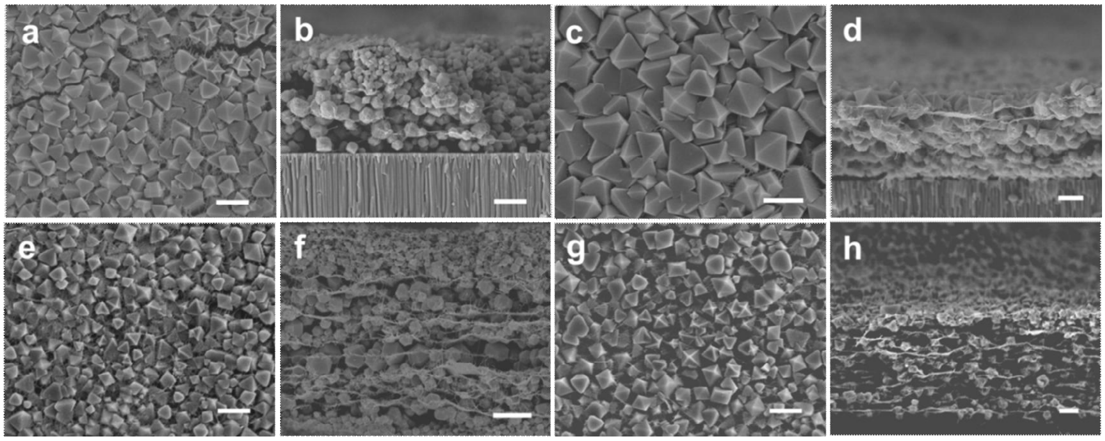

Supplementary Figure 7. Morphology of the HKUST-1/CNT thin films with different weight ratio of HKUST-1 to CNT. (a, b) 3:0.2; (c, d) 3:0.4; (e, f) 3:1.2; (g, h) 3:3. All the scale bars are 2 μm.

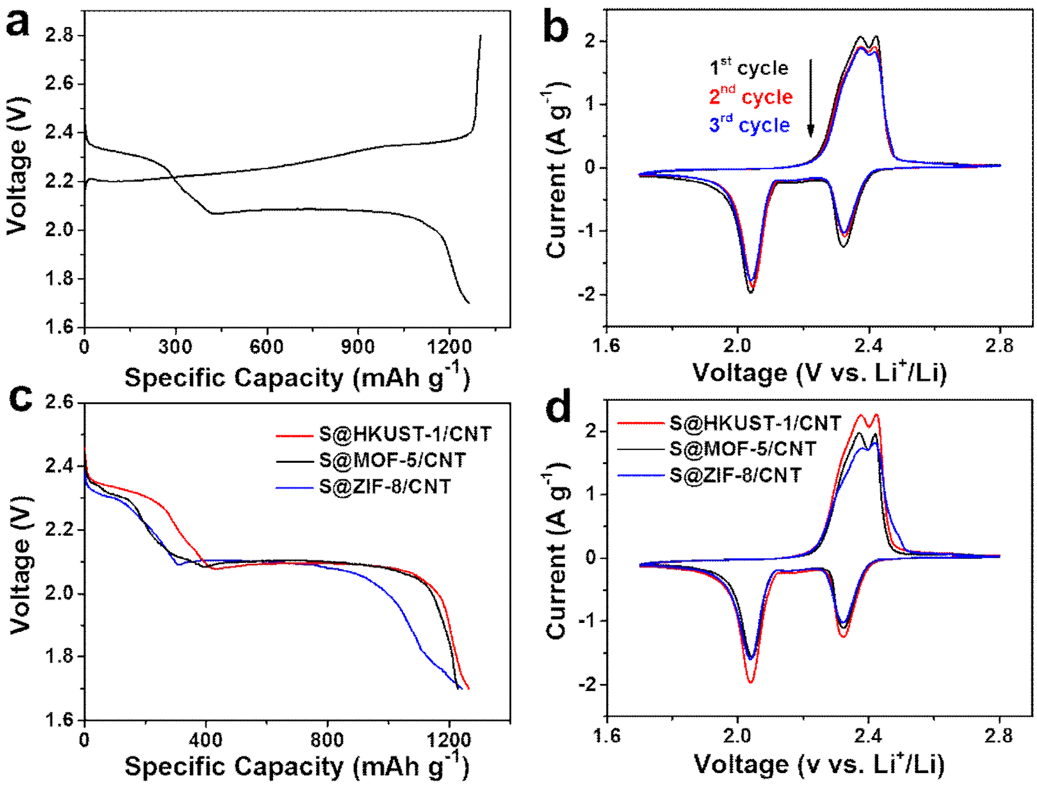

Supplementary Figure 8. Electrochemical characterization. (a) Charge-discharge profile and (b) CV curves of S@HKUST-1/CNT; (c) discharge profiles and (d) cyclic votammograms of S@HKUST-1/CNT, S@MOF-5/CNT and S@ZIF-8/CNT electrodes, respectively. The loading amount of sulfur is 1 mg cm^{-2}. The ratio of MOFs to CNT is 3:2.

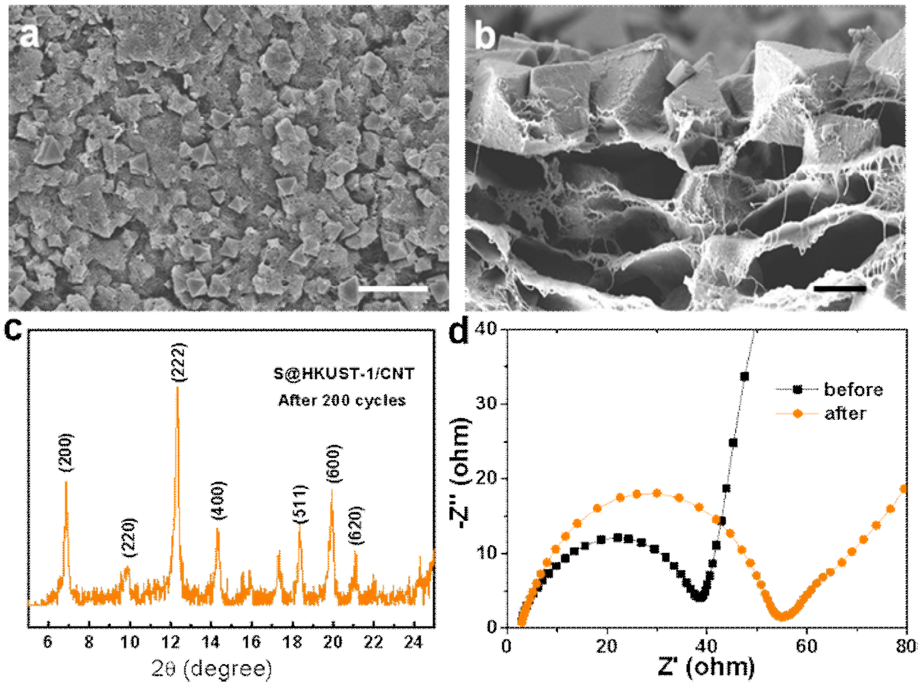

Supplementary Figure 9. Morphology and phase characterization of the S@HKUST-1/CNT with surfur loading of 1 mg cm^{-2} and the ratio of HKUST-1 to CNT of 3:2 after cycling 200 cycles. (a) Surface and (b) cross-section SEM images, (c) XRD and (d) EIS results of the flexible S@HKUST-1/CNT composite thin films after 200 cycles. The scale bars are 2 μm.

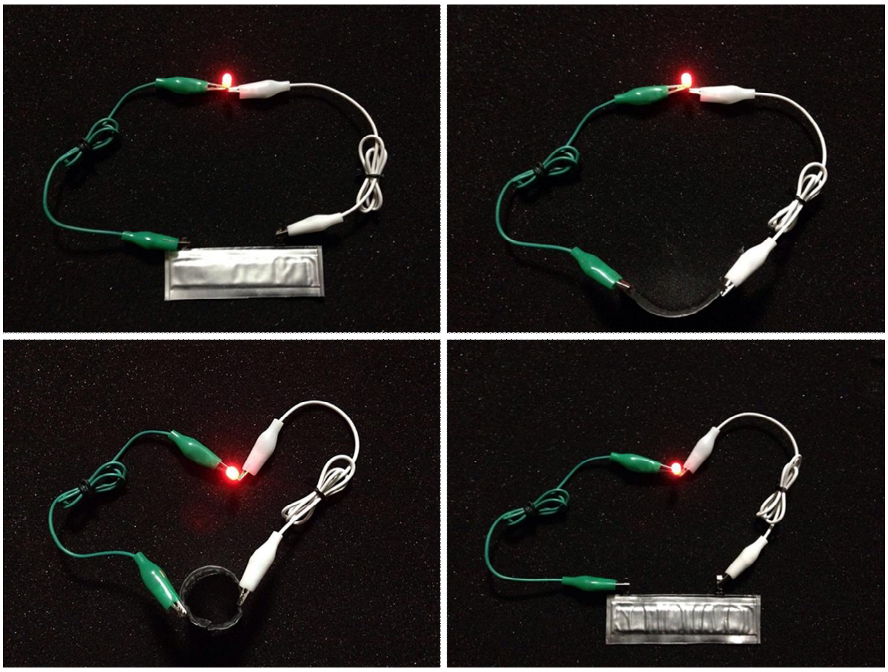

Supplementary Figure 10. The photographs of band shape soft package Li-S cell lighting up an LED in various bending extents. The cathode is S@HKUST-1/CNT with sulfur loading of 4.57 mg cm^{-2} with HKUST-1 to CNT ratio of 3:2.

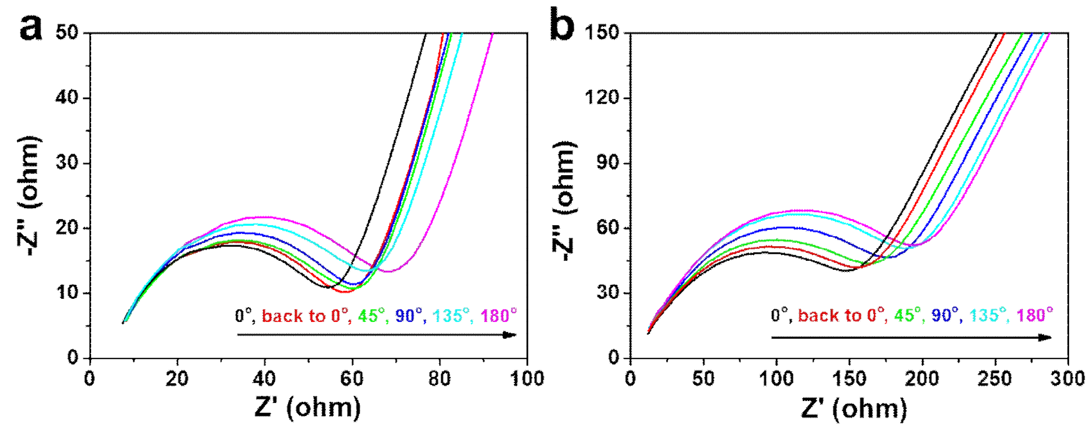

Supplementary Figure 11. The EIS study of the as-prepared soft package Li-S cell based on S@HKUST-1/CNT electrode with sulfur loading of 4.57 mg cm^{-2} and HKUST-1 to CNT ratio of 3:2 (a) before and (b) after 50 cycles under reversible foldings.

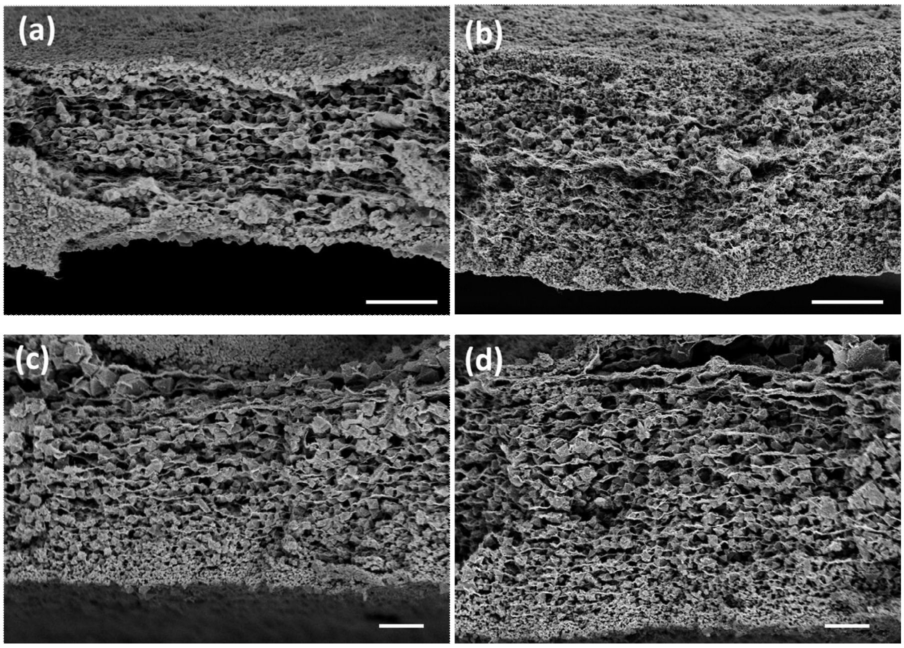

Supplementary Figure 12. Cross-section morphologies of S@HKUST-1/CNT thin films with different thicknesses of (a) 22.5, (b) 30.4, (c) 44.6, and (d) 64.7 μm. The scale bars are 10 μm.

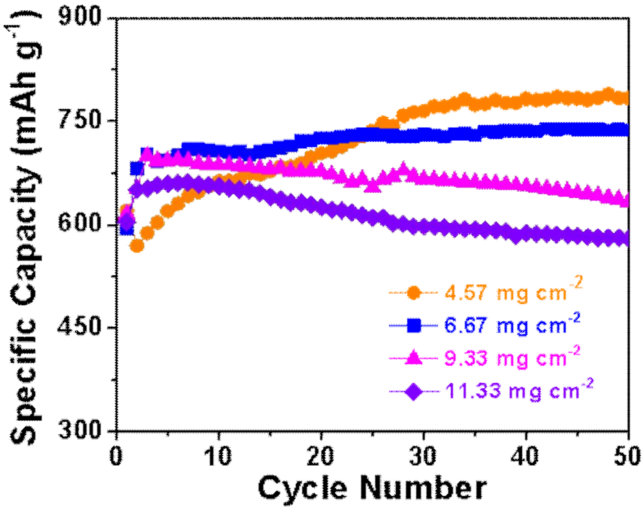

Supplementary Figure 13. The specific capacities of the S@HKUST-1/CNT electrodes with high sulfur loadings of 4.57, 6.67, 9.33, and 11.33 mg cm^{-2} cycling at 0.2 C up to 50 cycles.

Supplementary Table 1. The performance comparisons of MOFs-based sulfur electrodes.

Ref.	MOF	Rate	Maximum Capacity /mAh g^{-1}	Cycle Number	Capacity Retention /mAh g^{-1}	Rate Capacity /mAh g^{-1}
**This work**	**HKUST-1**	**0.2C**	**1263**	**100**<br>**200**<br>**300**<br>**500**	**911.4**<br>**851.3**<br>**782.6**<br>**680.5**	**10C, 449**
[1]	MOF-525	0.5C	1200	200	704	5C, 400
[2]	ZIF-8	0.5C	793	300	553	1C, 710
[3]	ZIF-8	0.1C	1200	200	420	1C, 450
[4]	ZIF-8	0.5C	735	250	590	-
[5]	MIL-101	0.1C	869	100	695	3C, 500
[6]	MIL-101	0.2C	980	50	650	-
[7]	Ni6(BTB)4(BP)3	0.1C	689	200	560	2C, 287
[8]	HKUST-1	0.05C	1498	170	500	-

The specific capacities and current rates are calculated based on elemental sulfur (1C = 1675 mA g^{-1})

Supplementary Table 2. Performance comparisons of the S@KHUST-1/CNT electrode with representative high-performance S@carbon cathodes.

Ref.	Sample	Sulfur loading /mg cm^{-2}	Rate	Cycle	Capacity/ mAh g^{-1}	Rate	Capacity/ mAh g^{-1}
**This work**	**S@HKUST-1-CNT**	**1.0**	**0.2C**	**100**<br>**200**<br>**300**<br>**500**	**911.4**<br>**851.3**<br>**782.6**<br>**680.5**	**10C**	**449**
9	S@Vertically Aligned Graphene Nanowalls	0.9	C/8	120	1210	8C	400
10	S@Crumpled N-Doped Graphene Sheets	1.5	0.46C	300	870	1C	950
11	S@Porous Gyroid Carbon	-	0.1C	100	420	1C	400
12	S@Hollow Core-shell interlinked Carbon Spheres	1.0	0.5C	200	950	6C	700
13	S@Amine-Functionalized Carbon Nanotubes	1.0	0.5C	300	750	4C	300
14	S@Mesoporous Carbon Nanotubes	-	0.1C	100	866	5C	847
15	S@Carbon Nanosheets	0.7-1.0	0.5C	500	612	4C	652
16	S@Hierarchical Porous Carbon	3.0-4.9	0.1C	80	875	2C	211
17	S@Hierarchical Porous Graphene	2.0	0.5C,	150	590	5C	656
18	S@Graphene Hybrid Nanosheets	-	0.5C	70	700	5C	350
19	S@Hollow Carbon Nanospheres	-	1C	500	629	2C	655
20	S@Multichannel Carbon Nanofibers	3.6	0.2C	200	950	2C	363
21	S@3D Porous Carbon	2.36	2C	1000	670	5C	500
22	Monodispersed Sulfur@rGO	-	0.5C	500	1017	4C	1089
23	S@Ketjenblack-MWCNT Sphere	2.5	0.1C	100	1207	0.5C	884
24	S@Aligned and Laminated Nanostructured Carbon Hybrid	1.0	2C<br>0.1C	1000<br>100	400<br>919	2C	473

Supplementary Table 2. continous

25	S@Microporous Carbon	0.2	0.24C	4000	475	2.85C	255
26	S@N-Doped Hollow Carbon Nanospheres	0.5-0.7	0.2C	100	980	2C	250
27	S@Ant-nest Carbon Structure	1.8	0.33C	200	700	3C	615
28	S@Hierarchical Carbon Nanotubes	0.8-1.0	1C	150	558	2C	491
29	S@Hollow Carbon	1.1-1.5	0.5C	300	720	9C	500
30	S@3D Vertically Aligned Carbon Sheets	1.0	0.5C	300	844	2C	738
31	S@MOF-derived Carbon Polyhedrons@rGO	1.0	0.18C	300	949	3C	479
32	S@Small CNTs Inside Large CNTs	1.36	1C	150	1146	2C	1274
33	S@3D N-rich CNTs@Grahpene	1.5	0.5C	200	896	5C	480
34	S@Si/SiO_2@Porous Carbon Sphere	1.5-1.7	2C	500	610	2C	614
35	S@B, N-rich Graphene	0.9-1.2	0.5C	500	556	3C	480
36	S@N-doped Hollow Porous Carbon Bowls	1.1-1.5	1	400	706	4C	535
37	S@Graphene quantum dots	1.0	0.5	100	1000	10C	540
38	S@Co, N-doped Graphitic Carbon	1.0-1.2	1	500	625	5C	565
39	S@Hollow Carbon Sphere@Polyeletrolyte @Graphene	1.0-1.3	0.6	200	575	2.4C	698

The active material loading, specific capacities and current rate are calculated based on elemental sulfur (1C = 1675 mA g^{-1})

Supplementary Table 3. Performance comparisons between S@HKUST-1/CNT with representative self-standing and/or flexible sulfur electrodes.

Ref.	Rate	Areal Sulfur loading/ mg cm^{-2}	Areal capacity/ mAh cm^{-2}	Electrode Thickness/ μm	Volumetric sulfur loading/ g cm^{-3}	Volumetric capacity/ Ah cm^{-3}
**This work**	**0.2C**	**11.33**	**6.57**	**80**	**1.41**	**0.82**
		**9.33**	**5.92**	**65**	**1.44**	**0.91**
		**6.67**	**4.92**	**45**	**1.48**	**1.09**
		**4.57**	**3.59**	**30**	**1.52**	**1.20**
[40]	0.2	2.5	2.2	140	0.18	0.16
[41]	0.1	7.0	5.2	200	0.35	0.26
[42]	0.5	6.8	4.8	80	0.85	0.60
[43]	0.1C	6.3	5.0	250	0.25	0.20
[44]	0.9C	1.25	0.71	50	0.25	0.14
[45]	0.1C	3.25	2.6	40	0.81	0.65
[46]	0.18C	2.45	2.57	50	0.49	0.51
[47]	0.1C	1.53	0.95	50	0.31	0.19
[48]	1.0C	2.3	1.9	50	0.46	0.38
[49]	0.1C	6.5	6.0	600	0.11	0.10
[50]	0.5C	2.0	2.2	90	0.22	0.24
[51]	0.06C	0.63	0.63	50	0.13	0.13
[52]	0.2C	3.9	3.86	120	0.33	0.32
[53]	0.2C	2.1	2.3	100	0.21	0.23
[54]	0.18C	10.1	9.9	1000	0.10	0.10
[55]	0.9C	1.8	1.53	70	0.26	0.22
[56]	0.37C	6.74	7.5	280	0.24	0.27
[20]	0.07C	10.8	8.0	210	0.51	0.38
[57]	0.2	3.2	3.84	60	0.53	0.64
[58]	0.25	1.6	1.84	70	0.23	0.26
[59]	0.5	3	2.55	150	0.2	0.17
[60]	0.2	9.8	6.86	1400	0.07	0.05
[61]	0.2	4.5	3.74	65	0.69	0.57
[62]	0.2	18.1	12.67	550	0.33	0.23
[63]	0.2	0.83	0.91	50	0.17	0.18
[64]	0.2	5.1	3.57	100	0.51	0.36

The areal capacity is valued by the 50th cycle. The active material loading, specific capacity, and current rate are calculated based on elemental sulfur (1C = 1675 mA g^{-1})

Supplementary Table 4. The EDX elemental content of S@MOFs/CNT electrode before and after CS_2 washing.

	S (wt%)	Cu (wt%)	Zn (wt%)	O (wt%)	C (wt%)	N (wt%)
S@HKUST-1/CNT	38.5	11.2	------	12.3	38.0	-----
S@HKUST-1/CNT washed by CS_2	34.2	12.1	------	13.3	40.2	-----
S@MOF-5/CNT	38.4	-----	11.2	13.3	37.1	-----
S@MOF-5/CNT washed by CS_2	31.1	-----	12.5	14.6	41.8	-----
S@ZIF-8/CNT	38.8	-----	10.8	------	40.8	9.6
S@ZIF-8/CNT washed by CS_2	5.0	-----	15.4	------	66.3	13.3

Supplementary Table 5. Summary of some critical performance data of obtained Li-S cells.

Cell configuration	Electrode	Sulfur loading (mg cm^{-2})	Sulfur content (wt %)	Specific capacity (mAh g^{-1})	thickness (μm)	Electrode capacity (mAh g^{-1})	Areal capacity (mAh cm^{-2})	Volumetric capacity (Ah L^{-1})
Coin cell	S@HKUST-1/CNT	1	40	988.5	22.5	395.4	0.99	439.3
	S@MOF-5/CNT	1	40	746.4	18.2	298.6	0.75	410.1
	S@ZIF-8/CNT	1	40	597.6	14.7	239.1	0.60	406.5
Soft package	S@HKUST-1/CNT	4.57	69.6	784.2	30.4	545.5	3.59	1194.6
		6.67	69.0	737.7	44.6	508.8	4.92	1093.4
		9.33	68.5	634.1	64.7	434.1	5.92	910.2
		11.33	68.2	579.7	80.0	395.0	6.59	821.0

The capacities are valued at the 50th cycle.

Supplementary References

1. Wang, Z., *et al.* Mixed-metal-organic framework with effective lewis acidic sites for sulfur confinement in high-performance lithium-sulfur batteries. *ACS Appl. Mater. Inter.* **7**, 20999–21004 (2015).
2. Zhou, J. W., *et al.* Rational design of a metal-organic framework host for sulfur storage in fast, long-cycle Li-S batteries. *Energy Environ. Sci.* **7**, 2715–2724 (2014).
3. Wang, Z., Dou, Z., Cui, Y., Yang, Y., Wang, Z. & Qian, G. Sulfur encapsulated zif-8 as cathode material for lithium–sulfur battery with improved cyclability. *Microporous Mesoporous Mater.* **185**, 92–96 (2014).
4. Zhou, J., *et al.* The impact of the particle size of a metal–organic framework for sulfur storage in Li–S batteries. *J. Mater. Chem. A* **3**, 8272–8275 (2015).
5. Zhao, Z. X., Wang, S., Liang, R., Li, Z., Shi, Z. C. & Chen, G. H. Graphene-wrapped chromium-MOF(MIL-101)/sulfur composite for performance improvement of high-rate rechargeable Li-S batteries. *J. Mater. Chem. A* **2**, 13509–13512 (2014).
6. Bao, W., Zhang, Z., Qu, Y., Zhou, C., Wang, X. & Li, J. Confine sulfur in mesoporous metal–organic framework @ reduced graphene oxide for lithium sulfur battery. *J. Alloys Compd.* **582**, 334–340 (2014).
7. Zheng, J., *et al.* Lewis acid-base interactions between polysulfides and metal organic framework in lithium sulfur batteries. *Nano Lett.* **14**, 2345–2352 (2014).
8. Wang, Z., *et al.* A metal–organic framework with open metal sites for enhanced confinement of sulfur and lithium–sulfur battery of long cycling life. *Gryst. Growth Des.* **13**, 5116–5120 (2013).
9. Li, B., Li, S., Liu, J., Wang, B. & Yang, S. Vertically aligned sulfur–graphene nanowalls on substrates for ultrafast lithium–sulfur batteries. *Nano Lett.* **15**, 3073–3079 (2015).
10. Song, J., Yu, Z., Gordin, M. L. & Wang, D. Advanced sulfur cathode enabled by highly crumpled nitrogen-doped graphene sheets for high-energy-density lithium-sulfur batteries. *Nano Lett.* **16**, 864-870 (2016).
11. Choudhury, S., *et al.* Nanoporous cathodes for high-energy Li-S batteries from gyroid block copolymer templates. *ACS Nano* **9**, 6147–6157 (2015).
12. Sun, Q., He, B., Zhang, X. Q. & Lu, A. H. Engineering of hollow core-shell interlinked carbon spheres for highly stable lithium-sulfur batteries. *ACS Nano* **9**, 8504–8513 (2015).
13. Ma, L., *et al.* Enhanced Li–S batteries using amine-functionalized carbon nanotubes in the cathode. *ACS Nano* **10**, 1050–1059 (2016).
14. Sun, L., *et al.* Sulfur embedded in a mesoporous carbon nanotube network as a binder-free electrode for high-performance lithium-sulfur batteries. *ACS Nano* **10**, 1300–1308 (2016).

15. He, B., Li, W. C., Yang, C., Wang, S. Q. & Lu, A. H. Incorporating sulfur inside the pores of carbons for advanced lithium-sulfur batteries: An electrolysis approach. *ACS Nano* **10**, 1633–1639 (2016).
16. Strubel, P., *et al.* ZnO hard templating for synthesis of hierarchical porous carbons with tailored porosity and high performance in lithium-sulfur battery. *Adv. Funct. Mater.* **25**, 287–297 (2015).
17. Tang, C., *et al.* CaO-templated growth of hierarchical porous graphene for high-power lithium-sulfur battery applications. *Adv. Funct. Mater.* **26**, 577–585 (2016).
18. Fei, L., *et al.* Graphene/sulfur hybrid nanosheets from a space-confined "sauna" reaction for high-performance lithium-sulfur batteries. *Adv. Mater.* **27**, 5936–5942 (2015).
19. Xu, F., *et al.* Facile synthesis of ultrahigh-surface-area hollow carbon nanospheres for enhanced adsorption and energy storage. *Nat. Commun.* **6**, 7221 (2015).
20. Li, Z., Zhang, J. T., Chen, Y. M., Li, J. & Lou, X. W. Pie-like electrode design for high-energy density lithium-sulfur batteries. *Nat Commun* **6**, 8850 (2015).
21. Li, G., Sun, J., Hou, W., Jiang, S., Huang, Y. & Geng, J. Three-dimensional porous carbon composites containing high sulfur nanoparticle content for high-performance lithium-sulfur batteries. *Nat Commun* **7**, 10601 (2016).
22. Chen, H., Wang, C., Dong, W., Lu, W., Du, Z. & Chen, L. Monodispersed sulfur nanoparticles for lithium-sulfur batteries with theoretical performance. *Nano Lett.* **15**, 798–802 (2015).
23. Ma, J., *et al.* Novel large-scale synthesis of a C/S nanocomposite with mixed conducting networks through a spray drying approach for Li-S batteries. *Adv. Energy Mater.* **5**, 1500046 (2015).
24. Sun, Q., *et al.* An aligned and laminated nanostructured carbon hybrid cathode for high-performance lithium-sulfur batteries. *Angew. Chem., Int. Ed.* **54**, 10539–10544 (2015).
25. Xu, Y., *et al.* Confined sulfur in microporous carbon renders superior cycling stability in Li/S batteries. *Adv. Funct. Mater.* **25**, 4312–4320 (2015).
26. Zhou, W. D., *et al.* Tailoring pore size of nitrogen-doped hollow carbon nanospheres for confining sulfur in lithium-sulfur batteries. *Adv. Energy Mater.* **5**, 1401752 (2015).
27. Ai, G., *et al.* Biomimetic ant-nest electrode structures for high sulfur ratio lithium-sulfur batteries. *Nano Lett.* **16**, 5365–5372 (2016).
28. Mi, K., Jiang, Y., Feng, J., Qian, Y. & Xiong, S. Hierarchical carbon nanotubes with a thick microporous wall and inner channel as efficient scaffolds for lithium-sulfur batteries. *Adv. Funct. Mater.* **26**, 1571–1579 (2016).
29. Li, M., *et al.* Gas pickering emulsion templated hollow carbon for high rate performance lithium sulfur batteries. *Adv. Funct. Mater.*, **26**, 8408–8417 (2016).
30. Rehman, S., *et al.* 3D vertically aligned and interconnected porous carbon nanosheets as sulfur immobilizers for high performance lithium-sulfur batteries. *Adv. Energy Mater.* **6**, 1502518 (2016).

31. Li, Z., *et al.* Reduced graphene oxide wrapped mofs-derived cobalt-doped porous carbon polyhedrons as sulfur immobilizers as cathodes for high performance lithium sulfur batteries. *Nano Energy,* **23**, 15–26 (2016).
32. Jin, F., Xiao, S., Lu, L. & Wang, Y. Efficient activation of high-loading sulfur by small cnts confined inside a large cnt for high-capacity and high-rate lithium-sulfur batteries. *Nano Lett.*, **16**, 440–447 (2015).
33. Ding, Y.-L., Kopold, P., Hahn, K., van Aken, P. A., Maier, J. & Yu, Y. Facile solid-state growth of 3D well-interconnected nitrogen-rich carbon nanotube-graphene hybrid architectures for lithium-sulfur batteries. *Adv. Funct. Mater.*, **26**, 1112–1119 (2015).
34. Rehman, S., Guo, S. & Hou, Y. Rational design of Si/SiO_2 @hierarchical porous carbon spheres as efficient polysulfide reservoirs for high-performance Li-S battery. *Adv. Mater.* **28**, 3167-3172 (2016).
35. Yuan, S. Y., Bao, J. L., Wang, L. N., Xia, Y. Y., Truhlar, D. G. & Wang, Y. G. Graphene-supported nitrogen and boron rich carbon layer for improved performance of lithium-sulfur batteries due to enhanced chemisorption of lithium polysulfides. *Adv. Energy Mater.* **6**, 1501733 (2016).
36. Pei, F., *et al.* From hollow carbon spheres to N-doped hollow porous carbon bowls: Rational design of hollow carbon host for Li-S batteries. *Adv. Energy Mater.*, **6**, 1502539 (2016).
37. Park, J., *et al.* Graphene quantum dots: Structural integrity and oxygen functional groups for high sulfur/sulfide utilization in lithium sulfur batteries. *NPG Asia Materials* **8**, e272 (2016).
38. Li, Y. J., Fan, J. M., Zheng, M. S. & Dong, Q. F. A novel synergistic composite with multi-functional effects for high-performance Li-S batteries. *Energy Environ. Sci.* **9**, 1998–2004 (2016).
39. Wu, F., *et al.* Layer-by-layer assembled architecture of polyelectrolyte multilayers and graphene sheets on hollow carbon spheres/sulfur composite for high-performance lithium-sulfur batteries. *Nano Lett.* **16**, 5488–5494 (2016).
40. Shi, J., Peng, H., Zhu, L., Zhu, W. & Zhang, Q. Template growth of porous graphene microspheres on layered double oxide catalysts and their applications in lithium–sulfur batteries. *Carbon* **92**, 96–105 (2015).
41. Cheng, X., *et al.* Three-dimensional aluminum foam/carbon nanotube scaffolds as long- and short-range electron pathways with improved sulfur loading for high energy density lithium–sulfur batteries. *J. Power Sources* **261**, 264–270 (2014).
42. Li, L., *et al.* A foldable lithium-sulfur battery. *ACS Nano* **9**, 11342–11350 (2015).
43. Yuan, Z., *et al.* Hierarchical free-standing carbon-nanotube paper electrodes with ultrahigh sulfur-loading for lithium-sulfur batteries. *Adv. Funct. Mater.* **24**, 6105–6112 (2014).
44. Zhou, G. M., *et al.* A flexible nanostructured sulphur-carbon nanotube cathode with high rate performance for Li-S batteries. *Energy Environ. Sci.* **5**, 8901–8906 (2012).

45. Jin, K. K., Zhou, X. F., Zhang, L. Z., Xin, X., Wan, G. H. & Liu, Z. P. Sulfur/carbon nanotube composite film as a flexible cathode for lithium-sulfur batteries. *J. Phys. Chem. C* **117**, 21112–21119 (2013).
46. Zhou, G., *et al.* A graphene-pure-sulfur sandwich structure for ultrafast, long-life lithium-sulfur batteries. *Adv. Mater.* **26**, 625–631 (2014).
47. Jin, J., *et al.* Flexible self-supporting graphene-sulfur paper for lithium sulfur batteries. *RSC Adv.* **3**, 2558–2560 (2013).
48. Huang, J. Q., *et al.* Flexible all-carbon interlinked nanoarchitectures as cathode scaffolds for high-rate lithium-sulfur batteries. *J. Mater. Chem. A* **2**, 10869–10875 (2014).
49. Elazari, R., Salitra, G., Garsuch, A., Panchenko, A. & Aurbach, D. Sulfur-impregnated activated carbon fiber cloth as a binder-free cathode for rechargeable Li-S batteries. *Adv. Mater.* **23**, 5641–5644 (2011).
50. Zhang, Z., Li, Q., Zhang, K., Chen, W., Lai, Y. & Li, J. Titanium-dioxide-grafted carbon paper with immobilized sulfur as a flexible free-standing cathode for superior lithium–sulfur batteries. *J. Power Sources* **290**, 159–167 (2015).
51. Wang, C., Wang, X., Wang, Y., Chen, J., Zhou, H. & Huang, Y. Macroporous free-standing nano-sulfur/reduced graphene oxide paper as stable cathode for lithium-sulfur battery. *Nano Energy* **11**, 678–686 (2015).
52. Zhou, G. M., Zhao, Y. B. & Manthiram, A. Dual-confined flexible sulfur cathodes encapsulated in nitrogen-doped double-shelled hollow carbon spheres and wrapped with graphene for Li-S batteries. *Adv. Energy Mater.* **5**, 1402263 (2015).
53. Wu, C., Fu, L., Maier, J. & Yu, Y. Free-standing graphene-based porous carbon films with three-dimensional hierarchical architecture for advanced flexible Li–sulfur batteries. *J. Mater. Chem. A* **3**, 9438–9445 (2015).
54. Zhou, G., *et al.* A graphene foam electrode with high sulfur loading for flexible and high energy Li-S batteries. *Nano Energy* **11**, 356–365 (2015).
55. Zhou, G., *et al.* A flexible sulfur-graphene-polypropylene separator integrated electrode for advanced Li-S batteries. *Adv. Mater.* **27**, 641-647 (2015).
56. Kim, J. S., Hwang, T. H., Kim, B. G., Min, J. & Choi, J. W. A lithium-sulfur battery with a high areal energy density. *Adv. Funct. Mater.* **24**, 5359–5367 (2014).
57. Zhou, G., Zhao, Y., Zu, C. & Manthiram, A. Free-standing TiO_2 nanowire-embedded graphene hybrid membrane for advanced Li/dissolved polysulfide batteries. *Nano Energy* **12**, 240–249 (2015).
58. Chen, H., *et al.* Rational design of cathode structure for high rate performance lithium-sulfur batteries. *Nano Lett.*, **15**, 5543–5548 (2015).
59. Kang, H.S. & Sun, Y.K. Freestanding bilayer carbon-sulfur cathode with function of entrapping polysulfide for high performance Li-S battery. *Adv. Funct. Mater.*, **26**, 1125–1132 (2015).
60. Hu, G., *et al.* 3D graphene-foam-reduced-graphene-oxide hybrid nested hierarchical networks for high-performance Li-S batteries. *Adv. Mater.*, **28**, 1603–1609 (2015).

61. Zhou, W., Guo, B., Gao, H. & Goodenough, J. B. Low-cost higher loading of a sulfur cathode. *Adv. Energy Mater.*, **6**, 1502059 (2015).
62. Qie, L., Zu, C. & Manthiram, A. A high energy lithium-sulfur battery with ultrahigh-loading lithium polysulfide cathode and its failure mechanism. *Adv. Energy Mater.* **6**, 1502459 (2016).
63. Wang, H., Zhang, W., Liu, H. & Guo, Z. A strategy for configuration of an integrated flexible sulfur cathode for high-performance lithium-sulfur batteries. *Angew. Chem. Int. Ed. Engl.* **55**, 3992–3996 (2016).
64. Chung, S. H., Chang, C. H. & Manthiram, A. Robust, ultra-tough flexible cathodes for high-energy Li-S batteries. *Small* **12**, 939–950 (2016).
